# Explosion pressure of industrial titanium powder and its suppression characteristics by explosion suppressants SiO_2_, K_2_CO_3_, and NaHCO_3_

**DOI:** 10.1038/s41598-024-83595-x

**Published:** 2024-12-30

**Authors:** Tianqi Liu, Kenan Liu

**Affiliations:** School of Safety Engineering, Shenyang Aerospace University, Shenyang, 110136 Liaoning China

**Keywords:** Powder explosion, Suppression effect, Maximum pressure, Explosion suppressant, Chemistry, Engineering

## Abstract

The ignition and explosion risks of industrial metal powder are significantly different from other types of powder, and its explosion suppression deserve attention. In this article, industrial titanium powder explosion is taken as the test object, and its explosion pressure and explosion suppression process are analyzed. The research results show that the mass concentration of powder clouds and ignition delay time have a great impact on the explosion pressure of titanium powder. The optimal concentration of powder cloud mass is 350 g m^−3^, under this condition, the rate of energy release per unit time is the highest, the maximum pressure of titanium powder explosion is 0.48 MPa. The optimal ignition delay time is 50 ms, changing the ignition delay time actually changes the state of the powder cloud at the ignition moment. The suppression effect can be ranked, and the best suppression agent is NaHCO_3_, followed by K_2_CO_3_, and finally SiO_2_. Under the effects of isolation, dilution, and cooling, NaHCO_3_ has the greatest effect on the explosion pressure of titanium powder.

## Introduction

There are many types of powder with explosive hazards in industry, and once these dusts explode during the production process, they can cause huge disasters^1–3^. Titanium metal is very common in modern industry. In 1791, the British first discovered this new metallic element in black magnetite. In 1795, German chemists also discovered this element while studying rutile and named it after the Greek god Titans. In 1910, American scientists first produced pure titanium by reducing TiCl_4_ with sodium. Subsequently, Luxembourg scientists reduced TiCl_4_ with magnesium to produce pure titanium. From then on, magnesium reduction method and sodium reduction method became industrial methods for producing sponge titanium. Titanium has excellent properties and has been widely used. Its application areas mainly include aviation and aerospace, shipbuilding, chemical and petrochemical, transportation, weapons, marine, electric power, construction, metallurgy, medical, sports equipment, daily necessities, and light industry. With the widespread use of industrial metal titanium, the explosion risk of titanium powder has gradually attracted widespread attention.

The hazards of industrial powder combustion and explosion have a significant impact on production. Current research indicates that agricultural grain powder, industrial coal dust, metal dust, and chemical dust can all cause explosion accidents, resulting in significant casualties^4–7^. Since the 21st century, industrial metal titanium has been increasingly valued by people, and its important position in production has also been constantly improving. Achievement on the combustion and explosion process of industrial powder has achieved some results, but the micro mechanism of powder explosion is currently unclear^8–15^. There are many reports on the research results of industrial powder explosion pressure and flame characteristics, which helps to understand the explosion propagation process and diffusion characteristics^16–25^. There are also many methods for studying powder explosions, and currently the main methods used are experiments and simulations^26–31^. Current research has found that the explosive power of industrial metal powder is much greater than that of other industrial powder. Titanium powder is prone to oxidation, combustion, and explosion, making it a hazardous material. The explosion of titanium powder poses a significant threat to people, property, and the environment. Therefore, the pressure change and explosion suppression of titanium powder explosion are of significance for preventing the ignition and combustion of titanium powder during production, transportation, and processing.

In addition to focusing on the characteristics of industrial powder explosions, current research has also conducted many reports on the suppression characteristics of industrial powder combustion^32–37^. The suppression of industrial powder explosion refers to mixing inert powder into industrial dust, greatly reducing the risk of explosion. There are many types of common combustion suppressants, including phosphates and carbonates, etc. Explosion suppressants can play many roles in controlling powder explosions, and the inhibitory effects of different powder combustion suppressants vary. Many explosion suppressants are the main components of industrial fire extinguishing agents^38–42^. From the perspective of explosion suppression mechanism, the mechanisms for suppressing industrial powder explosions include physical powder combustion suppression and chemical powder combustion suppression. The effect of chemical explosion suppression is usually much better than that of physical explosion suppression. However, chemical explosion suppressants are relatively expensive, so their cost-effectiveness is often not high. Therefore, it can be seen that there is still room for further research on the suppression of industrial powder combustion.

The authors of this paper have conducted some analysis on the combustion process and ignition feature of different industrial dust in the previous studies, and the propagation process and ignition feature of explosion flame have been obtained^43–53^. However, the current understanding of the explosion characteristics of titanium metal powder is not comprehensive, and there are also few related reports, which is also the innovation of this article. The aviation industry was the earliest department to develop and apply titanium and titanium alloys. Without titanium, it is impossible to manufacture supersonic aircraft for airplanes and engines. The application of titanium in the aerospace industry reduces launch weight, increases range, and saves costs, making it a popular material in the aerospace industry. Titanium powder is receiving increasing attention in industrial production, and the problem of titanium powder explosion is gradually becoming prominent. Therefore, it is necessary to study the explosion pressure and combustion suppression of titanium powder. This article will discuss the variation law of explosion pressure of titanium powder in enclosed spaces, as well as the inhibitory effect of a variety of inert powder on titanium powder explosions. Conducting research on the above-mentioned titanium powder explosion problem is crucial for mastering ignition prevention and control methods.

## Experimental process and preliminary preparation

### Experimental process and principles

**Fig. 1:**
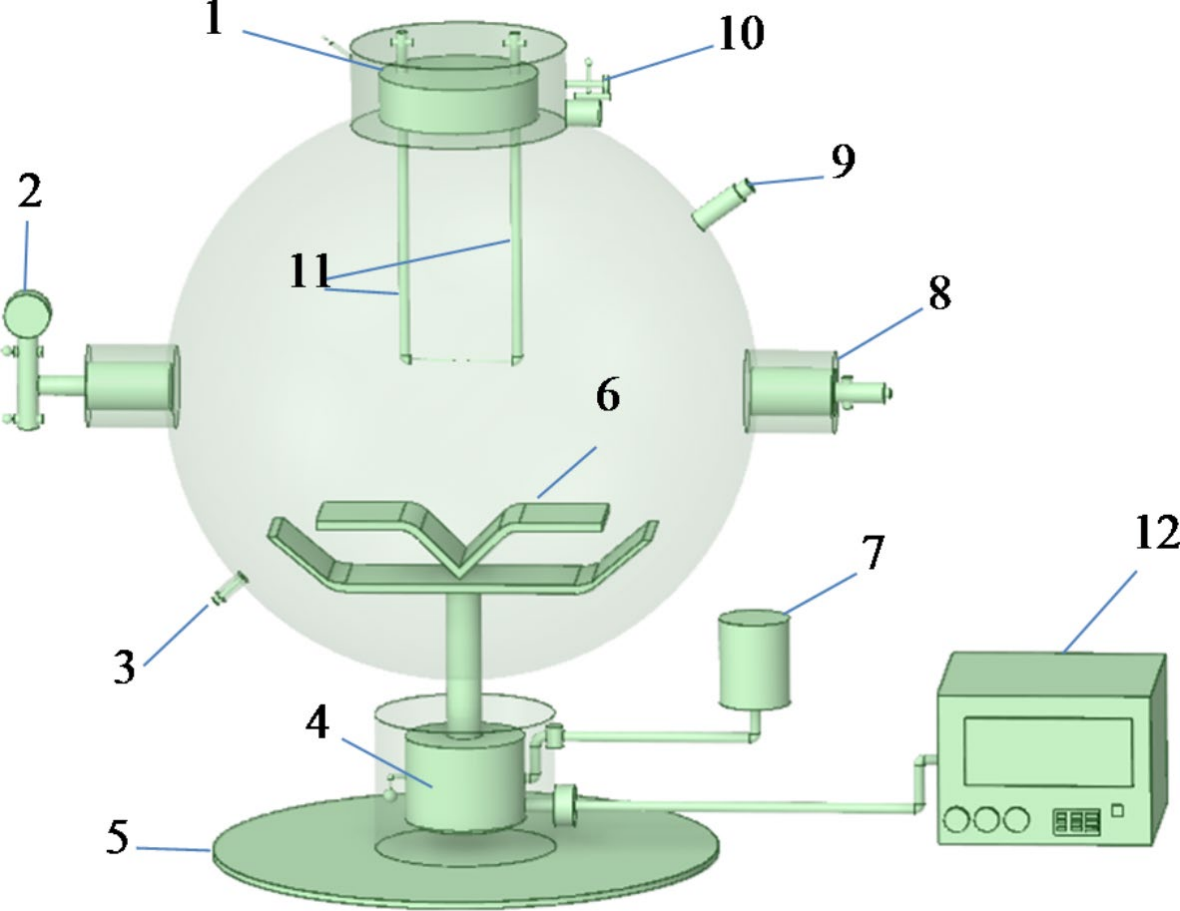
Powder explosion system. 1 Sealing cap; 2 vacuum gauge; 3 outlet of circulating water; 4 mechanical two-way valve; 5 base; 6 dispersion valve; 7 storage tank; 8 pressure sensor; 9 inlet of circulating water; 10 limit switch; 11 igniter; 12 console.

In Fig. 1, it is the experimental device used for powder explosion, which is drawn using Space Claim software. This experimental setup is a double-layer stainless steel structure. The ignition source of the experimental device is a chemical ignition head. The chemical ignition energy is 2 kJ. After continuous testing, this ignition energy is sufficient to ignite the dust used in the experiment, meeting the requirements for explosion. The experimental setup consists of three main parts: the main body of the device, the control system, and the data acquisition system. The main body of the device is an explosive ball. This device is currently internationally recognized, which means that the experimental results have reference value in different regions.

During the experiment, the screened powder is added to the powder storage tank at room temperature. A 20 L explosive ball is evacuated to -0.6 MPa, and the powder storage tank is filled with gas. The powder sample and air entered the tank to form a powder cloud, ensuring that the pressure inside the explosive ball is at atmospheric pressure before ignition. The ignition delay time is 60 ms, and a 2 kJ chemical ignition head is used to ignite at the center of the explosive ball. After the powder is ignited, the explosion pressure inside the container is detected by a pressure sensor installed on the wall of the container and transmitted to the data recording device. The pressure detection range of this experimental device is −0.1 ~ 2 MPa, the detection accuracy is 0.001 MPa, the data sampling interval is 0.2 ms, and the maximum sampling time is 12 s. The explosion situation can be determined through a circular quartz glass window, and then the pressure characteristic detection unit can detect pressure data and wirelessly transmit it to the data receiving end. The data can be transferred to a computer for further analysis. The maximum pressure *P*_max_ and the maximum pressure rise rate (d*P*/d*t*)_max_ of the titanium powder are obtained through the analysis of the pressure curve.

### Industrial titanium powder

Titanium is a transition metal element in the fourth group of the fourth period of the periodic table, with the element symbol Ti, atomic number 22, and relative atomic mass of 47.867. The physical and chemical properties of titanium metal are shown in Table 1. Titanium is a hard and shiny silver white metal. The purity of the titanium dust sample used in the experiment is 99.9%. The particle size distribution of titanium dust conforms to the Rosen Ramler function, and the median particle size is 75 μm. The relative density of titanium is 4.506, with a melting point of 1668 ℃ and a boiling point of 3287 ℃. The resistivity of titanium is 42 × 10^-8^ Ω m. Titanium in nature mainly exists in the form of rutile, perovskite, ilmenite, and other minerals. Titanium is widely distributed in nature, with its metal element content ranking seventh, only behind aluminum, iron, calcium, potassium, sodium, and magnesium. Titanium mainly exists in minerals in the form of titanium dioxide, titanate, and titanium silicate. Titanium has excellent properties and has been widely used. Its application areas mainly include aviation and aerospace, shipbuilding, chemical and petrochemical, transportation, weapons, marine, electric power, construction, metallurgy, medical, sports equipment, daily necessities, and light industry.

**Table 1:** Characteristic parameters of titanium metal samples.

Sample name	Physical property			Chemical property	
	Molecular weight	Density	Elongation rate	Boiling point	Melting point
Ti	47.867	4.506 g cm^−3^	50 ~60%	3287 ℃	1668 ℃

**Fig. 2:**
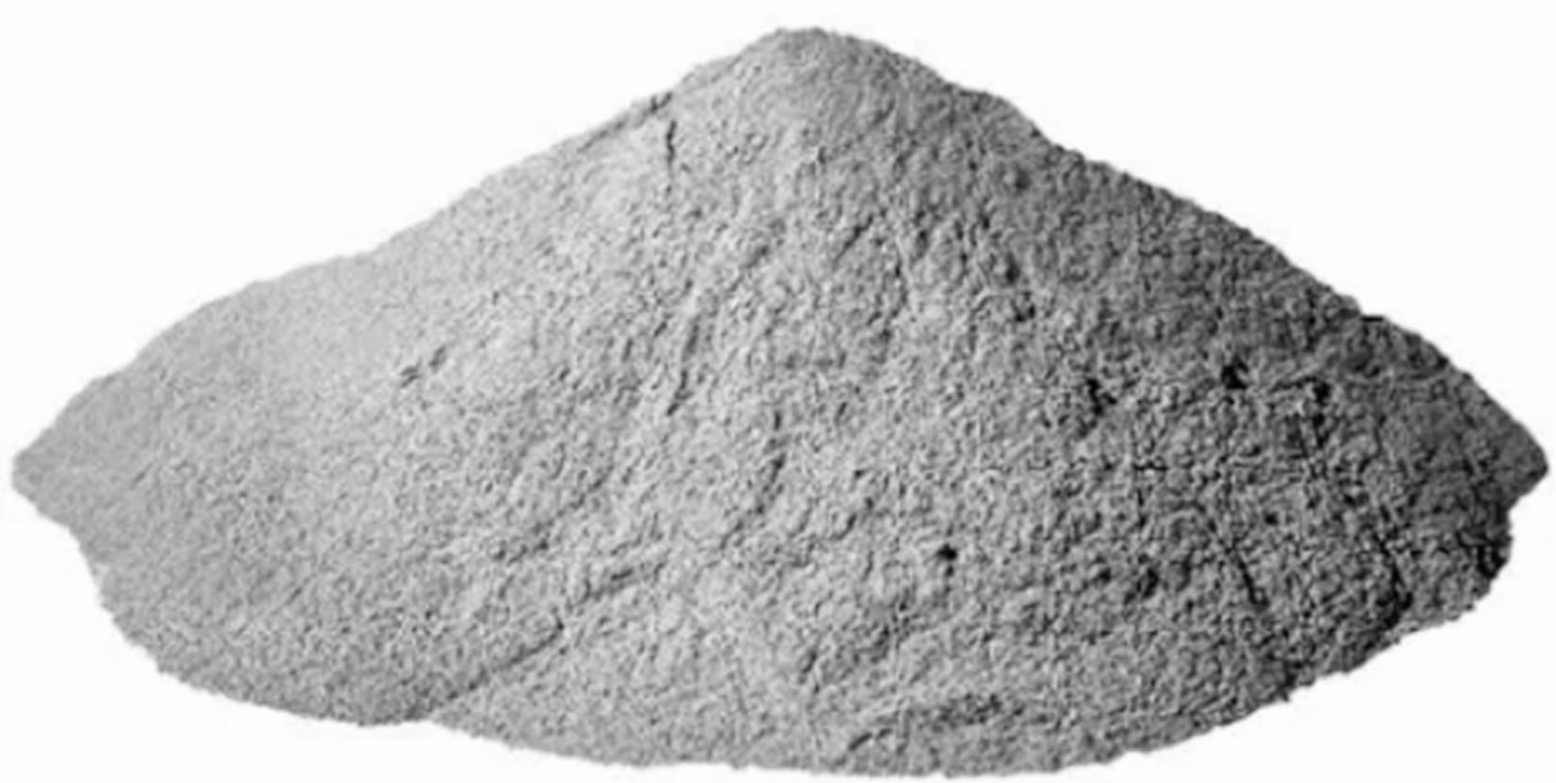
Industrial titanium powder sample.

In this study, the titanium powder samples used are shown in Fig. 2. Titanium has also been widely used in the aerospace industry to reduce launch weight, increase range, and save costs, making it a popular material in the aerospace industry. In the rocket, missile, and aerospace industries, it can be used as a pressure vessel, fuel storage tank, rocket engine shell, rocket nozzle sleeve, artificial satellite shell, manned spacecraft cabin, landing gear, lunar module, propulsion system, etc. Ilmenite is mainly distributed in Asia, Oceania, and Europe, with domestic ilmenite reserves ranking among the top. The top five countries with reserves are China (29.23%), Australia (24.62%), India (13.08%), Brazil (6.62%), and Norway (5.69%), with China and Australia accounting for more than half of the world’s reserves. Rutile is mainly distributed in North America, Africa, and Asia, with Australia accounting for 63.27% of the reserves. The top five countries with reserves are Australia (63.27%), India (15.10%), South Africa (13.27%), Ukraine (5.10%), and Mozambique (1.82%). China’s titanium and vanadium reserves rank first in the world, accounting for about 70% of the world’s total. China’s titanium vanadium ore resources are mainly distributed in western regions such as Sichuan, Yunnan, and Guangxi, with Panzhihua as the main storage area. Panzhihua has a total reserve of 898 million tons of titanium dioxide, of which 597.8 million tons are on balance sheet reserves, accounting for approximately 93% of the national reserves and 59% of the world reserves. During the mechanical treatment of titanium metal, a large amount of smoke and powder may be generated, and its explosion hazards are worth studying.

This study not only considers the explosion pressure of titanium metal powder, but also investigates the inhibitory effect of different ignition suppressants on titanium powder explosion. The three types of explosion suppressants used are SiO_2_, K_2_CO_3_, and NaHCO_3_. Silicon dioxide is an inorganic compound, a pure natural crystal of silicon dioxide, which is a hard, brittle, insoluble, colorless and transparent solid. Potassium carbonate is an inorganic substance that is easily soluble in water. The aqueous solution is alkaline and insoluble in ethanol, acetone, and ether. Sodium bicarbonate is an inorganic compound, white powder or fine crystal, odorless, salty, easily soluble in water, slightly soluble in ethanol, and slightly alkaline in aqueous solution. These three types of explosion suppressants are common materials for suppressing powder explosions, which is why they are chosen as explosion suppressants.

## Results and discussion

### Pressure changes of titanium powder cloud explosion in enclosed spaces

#### Relationship between titanium mass concentration and titanium powder explosion pressure

**Fig. 3:**
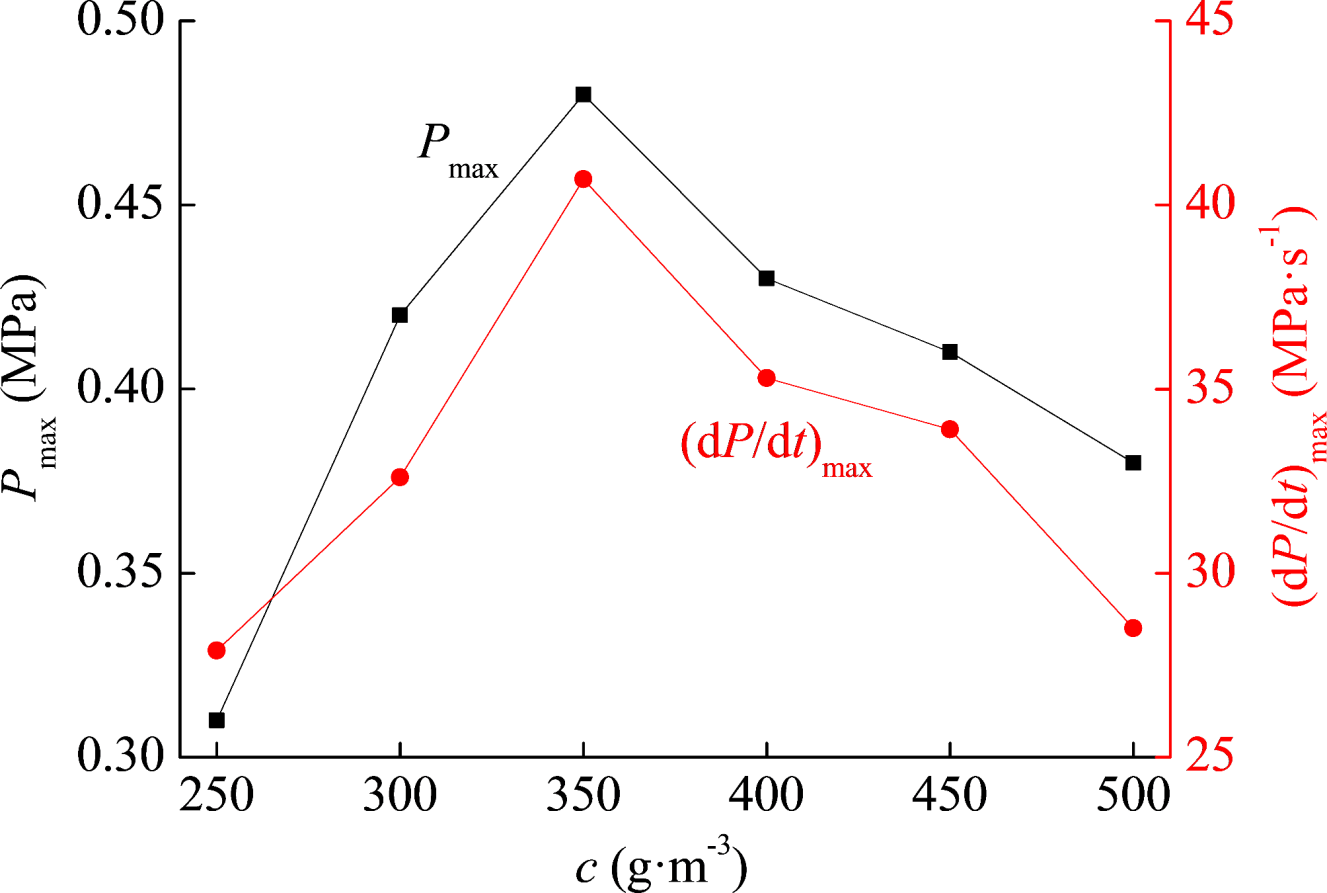
Pressure of titanium under different mass concentration.

The parameter settings for the metal titanium powder explosion experiment are as follows: ignition energy is 2 kJ, ignition delay time is 50 ms, and the median diameter of the powder sample is 75 μm. The particle size of titanium powder is selected as 75 μm, mainly because the explosion hazard of micron sized titanium powder is relatively high. If the median diameter is greater than 75 μm, it is not conducive to the release of explosive power, but if the particle size is too small, it is also not conducive to the increase of explosive power. Therefore, in this part, the selected median diameter is 75 μm. In the experiment of this part, considering that the mass concentration of titanium powder clouds can affect the titanium powder pressure, the mass concentration of titanium is constantly changed to obtain different explosion pressure data. In Table 2; Fig. 3, the results are given. The mass concentration of titanium is represented by the letter *c*, and the unit is g m^−3^. The experimental data clearly shows the relationship between pressure and titanium mass concentration.

**Table 2:** Pressure of titanium explosion under different mass concentration conditions.

c (g m^−3^)	250	300	350	400	450	500
*P*_max_ (MPa)	0.31	0.42	0.48	0.43	0.41	0.38
(d*P*/d*t*)_max_ (MPa s^−1^)	27.9	32.6	40.7	35.3	33.9	28.5

The mass concentration of titanium powder cloud has a significant impact on the pressure. Both *P*_max_ and (d*P*/d*t*)_max_ increase first and decrease later with the increase of *c*, indicating that *P*_max_ and (d*P*/d*t*)_max_ are consistent with the influence of *c*. The optimal mass concentration of titanium powder is 350 g m^−3^, indicating that under this condition, the dispersion state of powder cloud is optimal, and the rate of energy release per unit time is the highest, so the explosion pressure is also the highest. When the *c* of titanium powder cloud is 350 g m^−3^, the *P*_max_ of the titanium powder is 0.48 MPa, and the (d*P*/d*t*)_max_ of the titanium powder is 40.7 MPa s^−1^. At this time, the explosion intensity is the highest. If the *c* of titanium powder is too small, the release rate of explosion energy will decrease, and the explosion intensity will also decrease. If the *c* of titanium powder is too high, the oxygen concentration in the local space will not be sufficient, resulting in insufficient combustion process in a few quick seconds, so the intensity of the titanium explosion will also decrease.

#### Relationship between ignition delay time and titanium powder explosion pressure

**Fig. 4:**
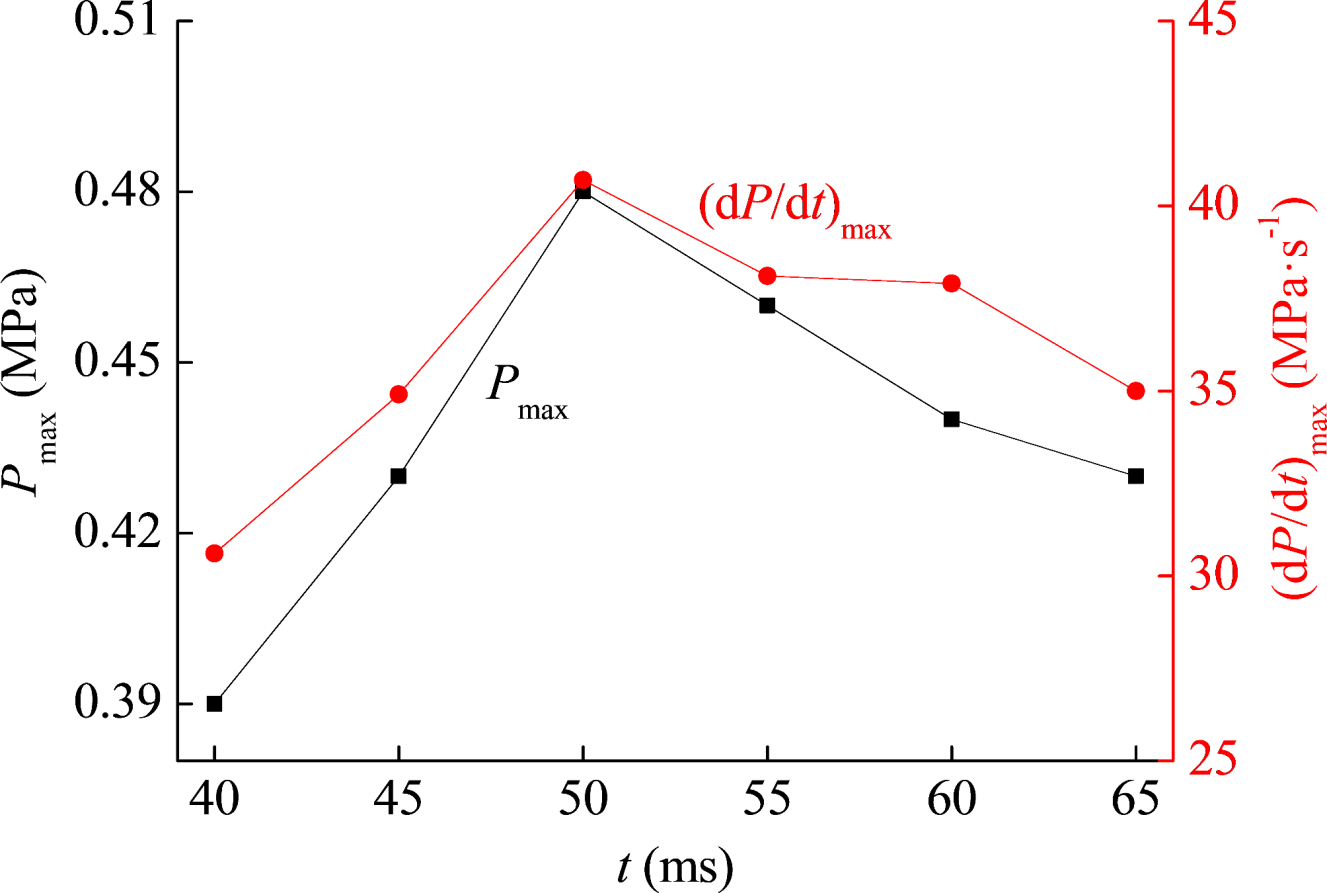
Explosion pressure of titanium powder cloud under different *t* conditions.

In addition to the mass concentration of titanium powder clouds affecting the explosion pressure, the ignition delay time also affects the explosion intensity. At present, we have obtained pressure change data under the condition of ignition delay time of 50 ms. By changing the ignition delay time and continuing the experiment, we can obtain explosion pressure data under different ignition delay time conditions. The results are shown in Table 3; Fig. 4. The powder cloud mass concentration selected for this set of explosion pressure experiment data is 350 g m^−3^. The ignition delay time is represented by the letter *t*, and its unit is ms. Through experimental data, it is evident that there is a close relationship between *t* and the *P*_max_ and (d*P*/d*t*)_max_ of titanium powder.

**Table 3:** Explosion pressure of titanium powder cloud under different *t* conditions.

t (ms)	40	45	50	55	60	65
*P*_max_ (MPa)	0.39	0.43	0.48	0.46	0.44	0.43
(d*P*/d*t*)_max_ (MPa s^−1^)	30.6	34.9	40.7	38.1	37.9	35.0

The ignition delay time is actually to control the pressure of the explosion based on the ignition conditions. Changing the ignition delay time actually changes the state of the powder cloud at the ignition moment. If the *t* is too long and the powder cloud does not fully diffuse near the ignition head, the explosion pressure will not be too high. If the *t* is too small, the particles of the powder cloud have not had time to settle down, and the explosion pressure will not be too high. So, there must be an optimal ignition delay time. According to the experimental data, it can be seen that the optimal *t* is 50 ms, because under this condition, the *P*_max_ and (d*P*/d*t*)_max_ of the titanium explosion are both maximum. As the *t* increases within 40 ~ 50 ms, the explosion pressure continues to increase. As the *t* increases within 50 ~ 65 ms, the explosion pressure decreases continuously.

### Suppression effect of suppressants SiO2, K2CO3, and NaHCO3 on pressure of titanium powder

**Fig. 5:**
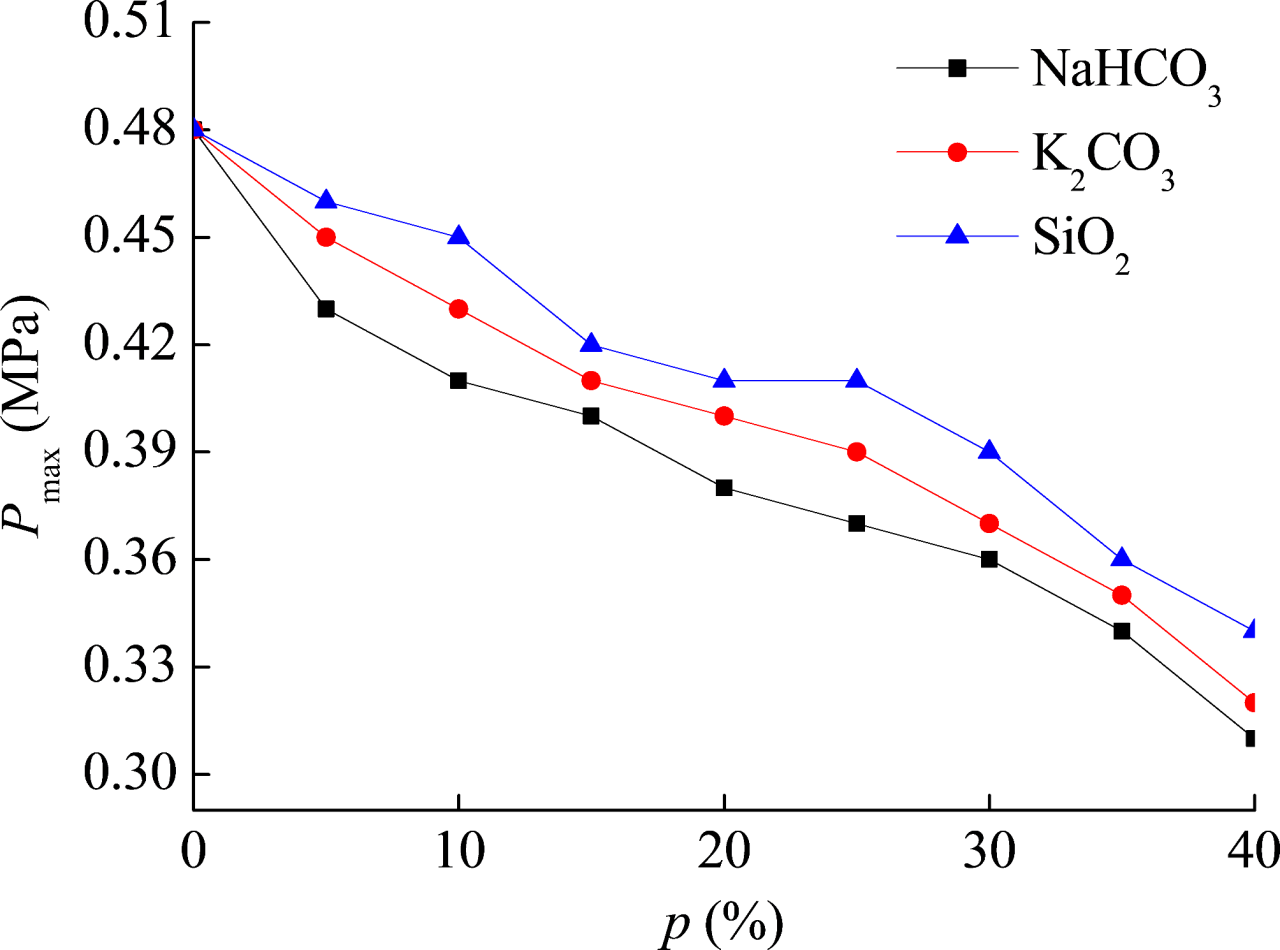
Suppression of different suppressants SiO_2_, K_2_CO_3_, and NaHCO_3_ on pressure of titanium powder.

Explosion suppression is a commonly used method in industry to prevent and control powder explosions, which can effectively control the spread of explosions. In this section of the study, the explosion suppressants used are SiO_2_, K_2_CO_3_, and NaHCO_3_. The purity of explosion suppressants is not less than 99.9%. The particle size of the selected suppressant is also consistent with that of titanium powder, with a median diameter of 75 μm. By mixing different explosion suppressants into titanium powder in different mass ratios, the inhibitory effect of the suppressant can be obtained. The *t* in the experiment is 50 ms, and the powder cloud mass concentration is set to 350 g m^−3^. The results of the suppression experiment are shown in Table 4; Fig. 5. The mass ratio of explosion suppressant added to titanium powder is represented by the letter *p*, and the unit is %. When conducting this part of the explosion suppression experiment, it is considered that the proportion of explosion suppression agent added cannot be too large, because in the actual explosion suppression process, the proportion of explosion suppression agent is often not very large, and the main body involved in the explosion should still be titanium powder. Therefore, during the experiment, the maximum mass proportion of explosion suppression agent added is 40%. The results obtained in this situation are also more informative.

**Table 4:** Suppression effect of suppressants SiO_2_, K_2_CO_3_, and NaHCO_3_ on pressure of titanium powder.

*p* (%)	*P*_max_ (MPa)			*p* (%)	*P*_max_ (MPa)		
	SiO_2_	K_2_CO_3_	NaHCO_3_		SiO_2_	K_2_CO_3_	NaHCO_3_
5	0.46	0.45	0.43	25	0.41	0.39	0.37
10	0.45	0.43	0.41	30	0.39	0.37	0.36
15	0.42	0.41	0.40	35	0.36	0.35	0.34
20	0.41	0.40	0.38	40	0.34	0.32	0.31

It can be clearly seen from the suppression experiment data that adding explosion suppressants can effectively suppress the explosion pressure of titanium powder. When the mass ratio of the added explosion suppressant is 40%, the maximum pressure of titanium powder explosion is less than 0.35 MPa, indicating that the explosion intensity of titanium powder has been greatly suppressed. According to the explosion pressure data of titanium powder under explosion suppression conditions, the suppression effect can be ranked, and the best suppression agent is NaHCO_3_, followed by K_2_CO_3_, and finally SiO_2_.

**Fig. 6:**
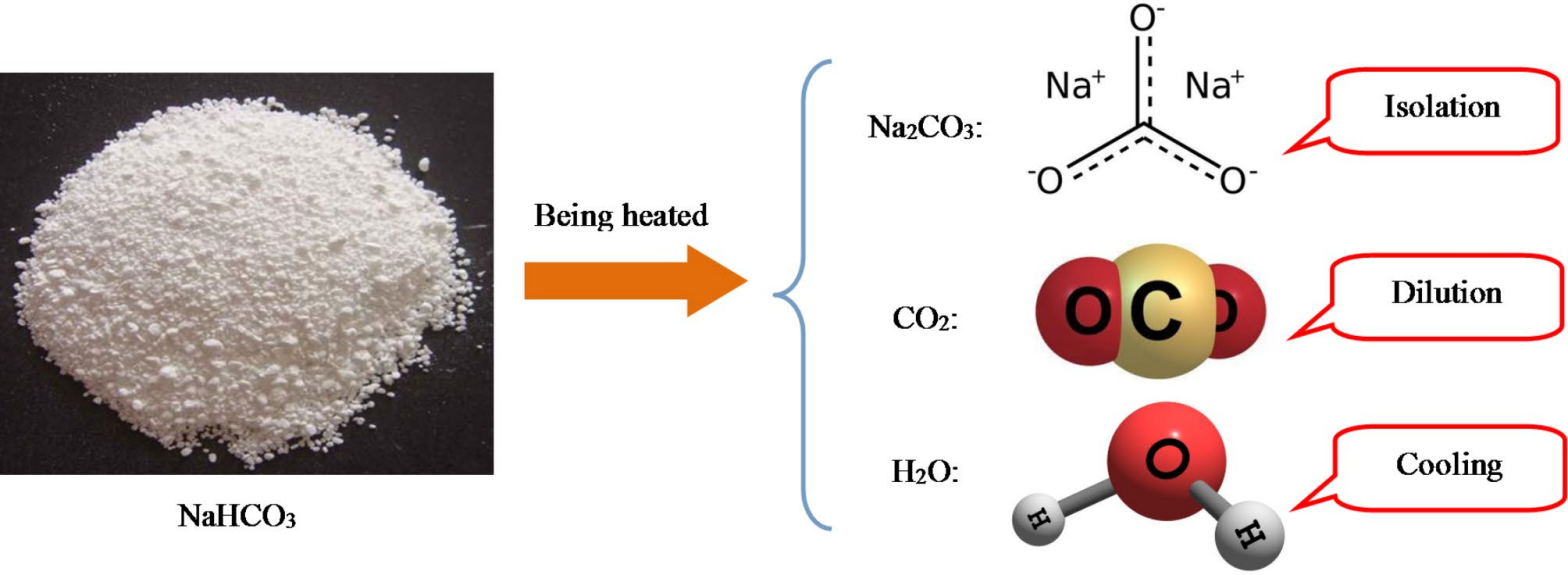
Analysis of suppression of NaHCO_3_ on titanium powder explosion.

Below is an analysis of the inhibition mechanisms of different explosion suppressants. The inhibitory mechanisms of different inhibitors are different. The explosion suppression mechanism mainly includes two types: physical suppression and chemical suppression. Inhibition of SiO_2_ is a typical physical inhibition process. And NaHCO_3_ inhibition belongs to a typical chemical inhibition process. In the process of chemical explosion suppression, there are often elements that are physically suppressed. The mechanism of chemical inhibition usually includes the control of explosion after the inhibitor undergoes a chemical reaction. The basic principles of explosion suppression include cooling principle, dilution principle, and isolation principle. A highly effective explosion suppressant can simultaneously exert multiple inhibitory effects. As shown in Fig. 6, the process of using a variety of suppressants to suppress titanium powder explosions is analyzed. Taking the explosion suppression process of NaHCO_3_ as an example for analysis, NaHCO_3_ will decompose into inert gases, generate inert solids, and also produce liquids that can cool the explosion. Under the combined action of these three suppression principles, the suppression of titanium explosion presents the best effect. The above research results have played a crucial role in the explosion prevention of industrial titanium powder.

## Conclusions

The relationship between the mass concentration of powder clouds and the explosion pressure of titanium powder clouds has been discussed. The results indicate that the maximum pressure and maximum pressure rise rate are consistent with the influence of powder cloud mass concentration. It is found that the optimal concentration of powder cloud mass is 350 g m^−3^.

The effect of ignition delay time on the explosion pressure of titanium powder cloud is analyzed. The optimal ignition delay time is 50 ms, under this condition, the dust cloud is suspended most evenly, and the intensity of titanium explosion is maximum. As the ignition delay time increases within 50 ~ 65 ms, the pressure decreases continuously.

The suppression on the pressure of titanium powder has been achieved. The best suppression agent is NaHCO_3_, followed by K_2_CO_3_, and finally SiO_2_. Inhibition of SiO_2_ is a typical physical inhibition process, and NaHCO_3_ inhibition belongs to a typical chemical inhibition process. NaHCO_3_ has inhibitory effects of isolation, dilution, and cooling simultaneously. The research results have important theoretical significance for the prevention and control of industrial titanium dust explosions.

## Data Availability

All data generated during this study are included in this published article.
